# Early argatroban and antiplatelet combination therapy in acute non-lacunar single subcortical infarct associated with mild intracranial atherosclerosis

**DOI:** 10.1186/s12883-021-02435-x

**Published:** 2021-11-10

**Authors:** Peng-Fei Wang, Zhuo-Ran Sun, Jin-Chao Yu, Na Geng, Ling-Yun Liu, Li-Na Zhu, Jing Li, Hai-Cheng Yuan, Guo-chen Zhao, Zhen-Guang Li

**Affiliations:** 1 Department of Neurology, Weihai Municipal Hospital, Cheeloo College of Medicine, Shandong University, No.70, Heping road, Huancui District, Weihai City, 264200 Shandong Province China; 2 Department of Neurology, Qingdao Central Hospital, Qingdao City, 266042 Shandong Province China; 3 School of Ocean Engineering, Harbin Institute of Technology at Weihai, Weihai, 264209 China

**Keywords:** Argatroban, Anticoagulation, Single subcortical infarction, Intracranial atherosclerosis, Early neurological deterioration

## Abstract

**Background:**

Patients with acute non-lacunar single subcortical infarct (SSI) associated with mild intracranial atherosclerosis (ICAS) have a relatively high incidence of early neurological deterioration (END), resulting in unfavorable functional outcomes. Whether the early administration of argatroban and aspirin or clopidogrel within 6–12 h after symptom onset is effective and safe in these patients is unknown.

**Methods:**

A review of the stroke database of Weihai Municipal Hospital, Cheeloo College of Medicine, Shandong University and Qingdao Center Hospital, Qingdao University Medical College in China was undertaken from May 2017 to January 2020 to identify all patients with non-lacunar SSI caused by ICAS within 6–12 h of symptom onset based on MRI screening. Patients were divided into two groups, one comprising those who received argatroban and mono antiplatelet therapy with aspirin or clopidogrel on admission (argatroban group), and the other those who received dual antiplatelet therapy (DAPT) with aspirin and clopidogrel during hospitalization (DAPT group). The primary outcome was recovery by 90 days after stroke based on a modified Rankin scale (mRS) score (0 to 1). The secondary outcome was END incidence within 120 h of admission. Safety outcomes were intracranial hemorrhage (ICH) and major extracranial bleeding. The probability of clinical benefit (mRS score 0–1 at 90 days) was estimated using multivariable logistic regression analysis.

**Results:**

A total of 304 acute non-lacunar SSI associated with mild ICAS patients were analyzed. At 90 days, 101 (74.2%) patients in the argatroban group and 80 (47.6%) in the DAPT group had an mRS score that improved from 0 to 1 (*P* < 0.001). The relative risk (95% credible interval) for an mRS score improving from 0 to 1 in the argatroban group was 1.50 (1.05–2.70). END occurred in 10 (7.3%) patients in the argatroban group compared with 37 (22.0%) in the DAPT group (*P* < 0.001). No patients experienced symptomatic hemorrhagic transformation.

**Conclusions:**

Early combined administration of argatroban and an antiplatelet agent (aspirin or clopidogrel) may be beneficial for patients with non-lacunar SSI associated with mild ICAS identified by MRI screening and may attenuate progressive neurological deficits.

**Trial registration:**

Our study is a retrospectively registered trial.

**Supplementary Information:**

The online version contains supplementary material available at 10.1186/s12883-021-02435-x.

## Background

Single subcortical infarctions (SSIs), are typically assumed to be caused by lipohyalinosis of a perforating artery [1]. However, increasing evidence has demonstrated that SSIs could also result from underlying large parent arterial atherosclerosis or a microatheroma in the proximal portion of a perforating artery [2, 3]. Treatment strategies and prognoses are differentiated based on the pathophysiology of individual SSI patients [4, 5]. Further, proximal SSIs associated with intracranial atherosclerosis (ICAS) lead to extended hospital stays and worse outcomes compared with distal SSI caused by lipohyalinosis of a perforating artery [6]. Although the combination of clopidogrel and aspirin appears to be superior to aspirin monotherapy for reducing the risk of stroke in the early stages of transient ischemic attack (TIA) or minor stroke [7], patients with SSI associated with intracranial atheromatous branch disease have progressive motor deficits and unfavorable functional outcomes [8]. Moreover, early neurological deterioration (END) occurs in ≥20% of SSI patients, hindering functional recovery, and is associated with an increased number of arterial stenosis and branch atheromatous lesions [9]. Therefore, new treatment approaches are required to prevent END and improve neurological deficits in patients with non-lacunar SSI associated with mild ICAS.

In recent decades, direct thrombin inhibitors (DTIs) have been developed with efficacy comparable to that of warfarin and are associated with significantly fewer bleeding complications [10]. Among the DTIs, argatroban can be administered early intravenously, directly inhibits free and clot-bound thrombin and thrombin-induced activities (such as platelet aggregation and endothelin-1 release), and is safe and feasible for use in the treatment of acute atherosclerosis-induced ischemic stroke [11]. Moreover, in patients with ischemic stroke associated with proximal intracranial arterial occlusion treated with recombinant tissue plasminogen activator (r-tPA), adjuvant argatroban use resulted in excellent outcomes at 90 days and was not associated with an increased risk of symptomatic intracerebral hemorrhage (ICH) [12]. These results suggested that argatroban may be effective and safe for treating acute atherosclerotic ischemic stroke.

The aim of the present study was to investigate whether the early administration of argatroban and aspirin or clopidogrel in patients with non-lacunar SSI associated with mild ICAS within 6–12 h after symptom onset is effective and safe by analyzing data from the stroke database of Weihai Municipal Hospital, Cheeloo College of Medicine, Shandong University and Qingdao Center Hospital, Qingdao University Medical College in China.

## Methods

### Study design and inclusion/exclusion criteria

This was a retrospective, observational cohort study using data from the stroke database of Weihai Municipal Hospital, Cheeloo College of Medicine, Shandong University and Qingdao Center Hospital, Qingdao University Medical College, two tertiary hospitals in Shandong Province, China. The database contains the following information for each patient: demographics, diagnosis, CT/MR information, laboratory and color Doppler ultrasound results, outcomes, drugs used, and disease-specific data. For patients with stroke, National Institutes of Health Stroke Scale (NIHSS) and modified Rankin scale (mRS) scores are also documented. These scores are evaluated at admission, during the hospital stay, at discharge, and at 90 days after stroke by telephone follow-up with the attending physicians who are independent and proficient in these assessments. These scores are entered as clinical data for each patient. Functional independence was defined as an mRS score ≤ 1.

The study protocol was separately approved by Weihai Municipal Hospital, Shandong University ethics committee and Qingdao Central Hospital ethics committee. Because this was a retrospective observational cohort study, the data were anonymous; consequently, the ethics committee waived the requirement for informed consent. All methods were performed in accordance with the relevant guidelines and regulations.

The inclusion criteria were as follows: (i) Age between 40 and 80 years; (ii) NIHSS score ≥ 2 with SSI in the middle cerebral artery (MCA) territory according to the Stop Stroke Study Trial of Org 10172 in Acute Stroke Treatment (SSS-TOAST) classification criteria [13]; (iii) < 50% MCA stenosis on magnetic resonance angiography (MRA); and (iv) lesion location in the lowest portion of the basal ganglia and infarction diameter > 15 mm [4].

Patients were excluded if they (i) were physically or subjectively unable to comply with MRI; (ii) exhibited cardioembolic risk factors (atrial fibrillation [AF], valvular heart disease, postcardiac valve replacement, etc.); (iii) had ≥50% stenosis of the ipsilateral carotid artery; (iv) presented with ischemic stroke with other or undetermined causes; (v) were in a stupor or coma; (vi) had cancer or any other severe concurrent disease; or (vii) were pregnant.

The included patients were divided into two groups, namely, those receiving argatroban (Lunan Pharmaceutical Group, Shandong, China) and monoantiplatelet therapy with aspirin or clopidogrel on admission (argatroban group) and those receiving dual antiplatelet therapy with aspirin and clopidogrel during hospitalization (DAPT group). In the argatroban group, argatroban was continuously infused at 60 mg/day during the first 2 days, and then twice a day (20 mg/day) for 5 days. Meanwhile, aspirin (100 mg/day) or clopidogrel (75 mg/day) was also administered. In the DAPT group, aspirin (100 mg/day) and clopidogrel were given at an initial dose of 300 mg, followed by 75 mg per day for 3 weeks. Subsequently, the patients took either aspirin or clopidogrel. All patients received basic stroke care, including statin treatment, flat head positioning, oxygen, swallowing assessment, and compression stockings or devices, unless contraindicated. Antihypertensive agents were administered when the systolic pressure exceeded 185 mmHg. Systolic pressure was maintained at 130–140 mmHg [14]. In our study, the initial treatment of argatroban with a single antiplatelet agent or DAPT were mainly based on the experience and judgement of the attending physician for each patient.

### Data collection and processing

Data for demographics; pre-existing comorbidities; concomitant therapies; activated partial thromboplastin time (APTT) and magnetic resonance imaging (MRI) data on admission; hospitalization days; drugs used during hospitalization; and NIHSS and mRS scores at admission, during hospitalization, at discharge, and 90 days after stroke were extracted for each patient. The preexisting comorbidities included hypertension, dyslipidemia, diabetes mellitus, heart failure, chronic renal failure, and previous stroke. Previous stroke was identified as a history of ischemic stroke. The MRI data included initial infarction size and the presence of microbleeds and plaques on the superior side of the MCA. Venous thrombosis of the lower extremities was diagnosed by color Doppler ultrasound.

### MRI and HR–MRI analysis

A total of 304 SSI patients underwent MRI on a 3.0 T MR scanner (Magnetom Skyra, Siemens Healthcare, Germany). The imaging sequences obtained included three-dimensional time-of-flight MRA (3D TOF–MRA) (repetition time, 21 ms; echo time, 3.43 ms; flip angle, 18°; slice thickness, 0.5 mm); axial T2-weighted images (repetition time, 4000 ms; echo time, 95 ms); T1-weighted images (repetition time, 180 ms; echo time, 2.5 ms); a fluid-attenuated inversion recovery sequence (repetition time, 7500 ms; echo time, 95 ms); and diffusion-weighted images (repetition time, 3400 ms; echo time, 93 ms). Except for MRA, all the above sequences had a 5-mm slice thickness and a 1.5-mm interslice gap. MR images were viewed using Picture Archiving and Communication Systems software (Medcare, China). Intracranial vessels were examined by 3D TOF MRA and extracranial carotid arteries were examined by duplex color Doppler ultrasound or contrast-enhanced MRA.

A total of 78 patients underwent high-resolution (HR) MRI, and images of the main trunk of the MCA supplying the infarcted region were acquired using 3.0 T HR–MRI (Magnetom Skyra). Imaging sequences obtained included a 2D T2WI sequence (repetition time, 4000 ms; echo time, 62 ms; flip angle, 150°; slice thickness, 2 mm without an interslice gap) and a 3D T1-weighted sampling perfection with application-optimized contrasts using different flip angle evolution sequence (repetition time, 700 ms; echo time, 15 ms; spatial resolution, 0.6 × 0.6 × 0.6 mm). Plaques were identified based on the presence of eccentric wall thickening, while thin sections were those estimated to have < 50% of the thickness of the thickest point by visual inspection. The locations of atherosclerotic plaques were classified as being centered on the superior side (the usual origin of MCA perforators) or the inferior side of the vessel [15].

All brain MR images were evaluated in a blind manner by two physicians with advice from a third experienced physician in case of disagreement.

### END definition and data collection

All patients were admitted to the stroke unit. The NIHSS score of every stroke patient was rated by attending physicians every 12 h and at least once a day in the stroke unit. END was defined as neurological deterioration occurring within 120 h of admission. In this study, END cases were defined using the following criteria: (1) An increase of ≥2 points in the total NIHSS score, (ii) an increase in the motor score (5a–6b) of ≥1 point, or (iii) any new neurological deficit (including deficits that were not measurable by the NIHSS score) [9]. Considering the heterogeneity of END assessment in medical records, our third-party assessment team, blinded to the study group assignment and treatment, conducted a central assessment of END based on medical records. If the assessment results were inconsistent with the previous assessment, and the third assessor was intervened in the assessment. When patients were diagnosed as END in each treatment group, the volume expansion treatment, intensive statin and the rehabilitation therapy were administered [14, 16].

### Outcomes

The primary outcome was recovery of the mRS score from 0 to 1 by 90 days after stroke. The secondary outcome was END incidence within 120 h of admission. The NIHSS scores at discharge, the occurrence rate of venous thrombosis in the lower extremities, hospitalization days, the APTT at 24 h, and the location of atherosclerotic plaques on the superior side of the MCA were also assessed. Safety outcomes were ICH and major extracranial bleeding during hospitalization. Major extracranial bleeding included gastrointestinal bleeding, urinary bleeding, skin mucosal bleeding, and gingival bleeding.

### Statistical analyses

Mean (with standard deviation) or median (interquartile range [IQR]) was used to describe continuous variables relating to patient characteristics and were compared by independent sample T tests. Categorical variables were described as frequencies (percentages) and were compared by the χ^2^ test. Univariable analysis was first performed to identify potential factors associated with good functional outcomes (mRS score 0–1). Binary logistic regression with the ‘Enter’ method was used to identify independent factors for good functional outcome. SPSS version 20.0 for Windows was used for statistical analysis. The minimum patient sample size required was determined to be N1 = 147 and N2 = 118 according to the available trial results.

## Results

**Fig. 1:**
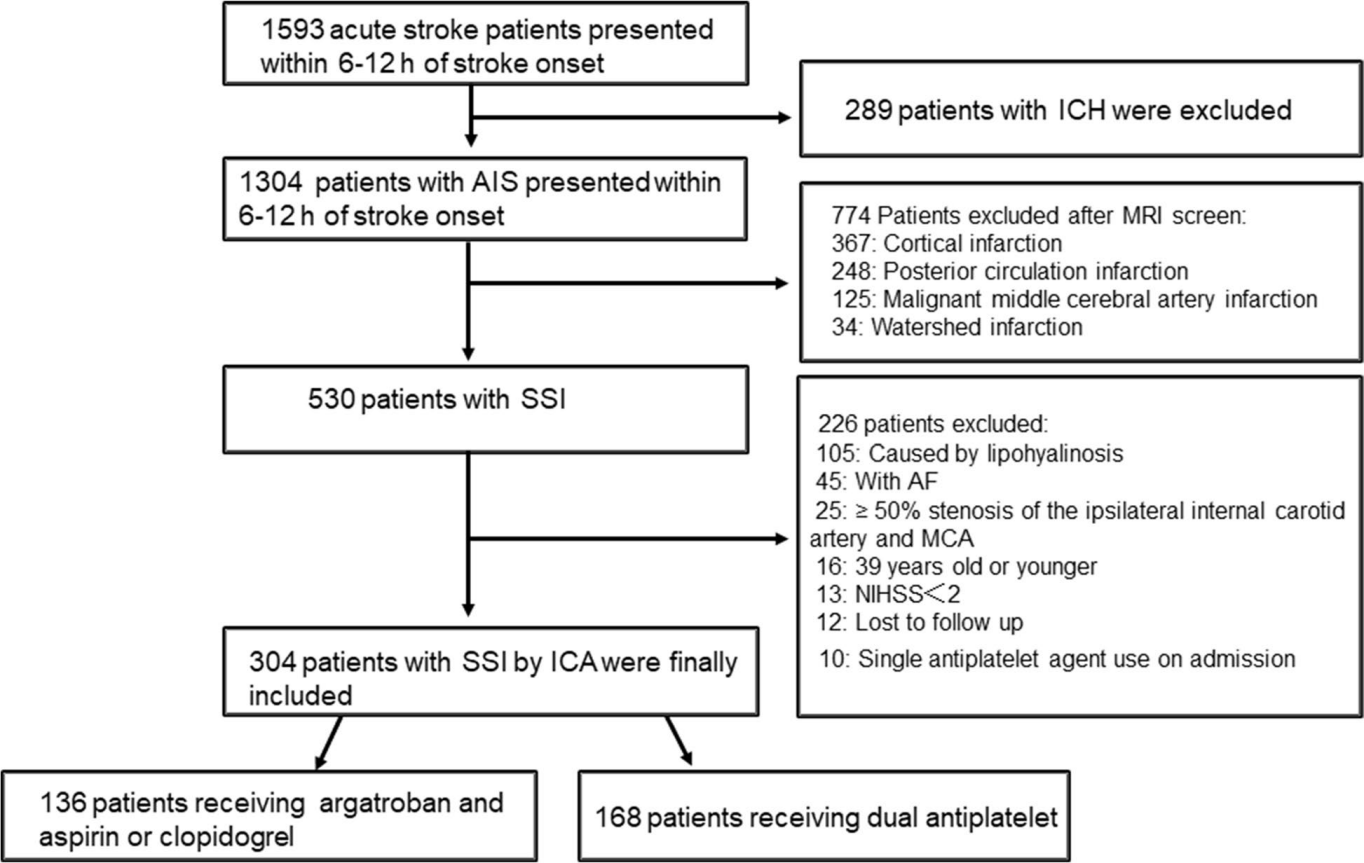
Patient flow diagram

From May 2017 to January 2020, a total of 1593 patients with acute stroke were admitted within 6–12 h after stroke onset. After excluding those with ICH, 1304 patients with ischemic stroke were available for further analysis. After MRI screening, 530 patients were identified as having an SSI, while an additional 226 patients were excluded for the following reasons: SSI caused by lipohyalinosis; exhibiting AF; ≥50% stenosis of the ipsilateral internal carotid artery and/or MCA; under 40 years of age; initial NIHSS score < 2; lost to follow-up; or the use of a single antiplatelet agent on admission. Finally, 304 patients (136 patients in the argatroban group and 168 in the DAPT group) with non-lacunar SSI associated with mild ICAS were included for analysis in this study. Among them, 3 patients in the argatroban group received argatroban for less than 7 days and 2 patients in the DAPT group received DAPT for less than 21 days. All of these patients were included into analysis. The flow diagram of the study cohort is shown in Fig. 1.

### Confirmation of non-lacunar SSI associated with mild ICAS

**Fig. 2:**
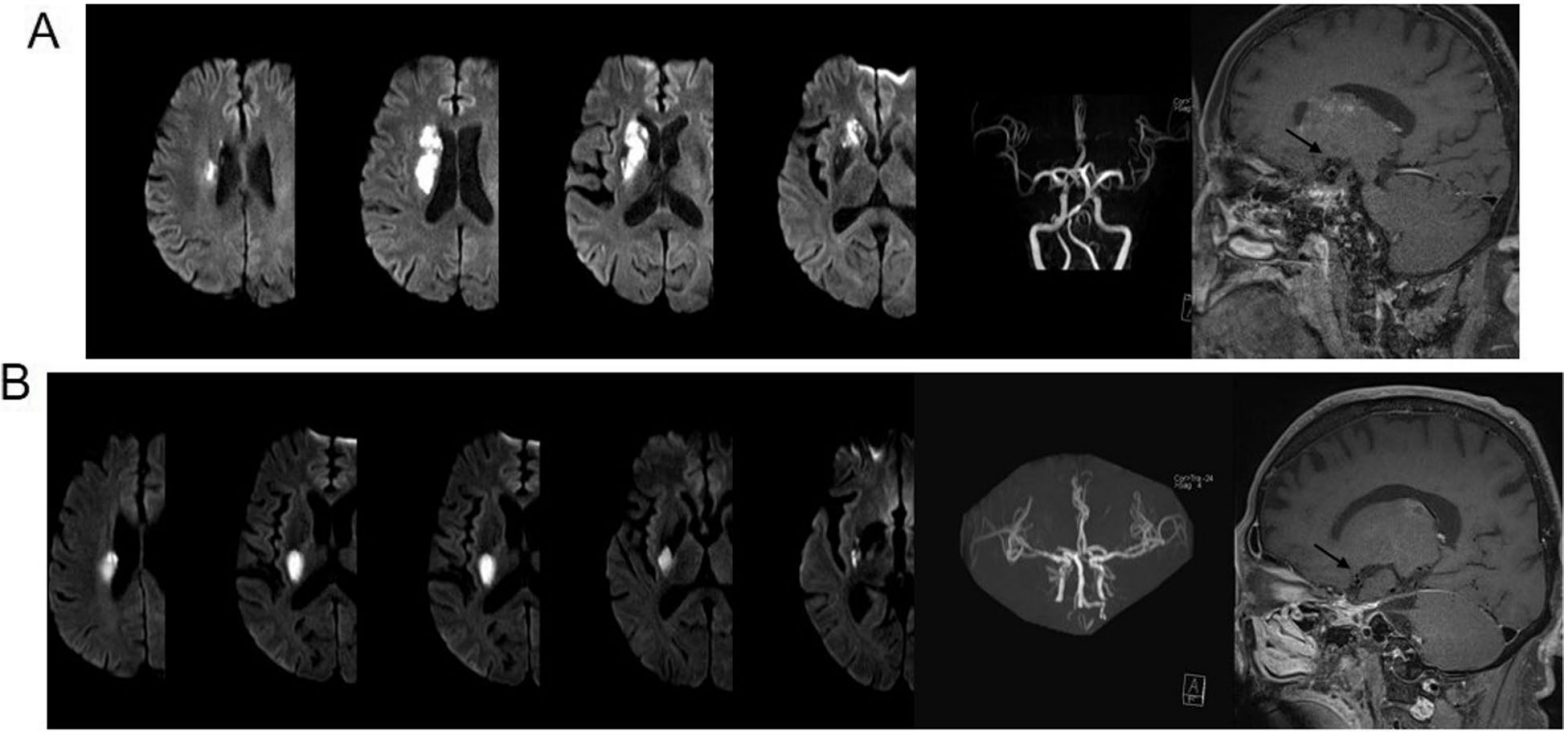
Example of SSI with ICAS in the MCA territory. Involvement of the lowest portion of the basal ganglia was considered an extension to the basal surface of the parent artery, and an atheromatous plaque is shown at the superior portion of the MCA. The black arrow represents the plaque

All patients with SSI underwent 3.0-T MRI + MRA, while 78 underwent HR–MRI for evaluation of plaques on the vessel wall. Cases of non-lacunar SSI associated with mild ICAS were confirmed based on the lesion location in relation to the lowest portion of the basal ganglia and the MCA parent artery, and infarction with a diameter > 15 mm and exceeding > 3 imaging slices [1, 3] (Fig. 2).

### Baseline characteristics in the argatroban and DAPT groups

As shown in Table 1, there were no differences between the two groups with respect to sex, age, hypertension, diabetes mellitus, hyperlipidemia, coronary artery disease, current smoking habits, previous history of stroke, initial infarction diameter, or the presence of microbleeds. The hematocrit, fibrinogen level, white blood cell (WBC) count, renal function, blood glucose level, statin use, and antihypertensive use did not differ between the two groups; however, the systolic blood pressure (SBP) was lower in patients of the argatroban group than in those of the DAPT group. The initial NIHSS scores in the argatroban group were higher than those in the DAPT group but were not significantly different. In the argatroban group, 62.5% of the plaques were located on the superior side of the MCA compared with 54.3% for the DAPT group. Although the difference was not statistically significant, these data suggested that plaques on the superior side are associated with SSI caused by ICAS (Table 1). However, Age, diabetes mellitus, glucose levels, SBP, initial infarction diameter, percentage of plaques on the superior side, and argatroban group were shown to be significantly different between mRS 0–1 and mRS 2–6 group (Supplementary Table S1).

**Table 1:** Baseline characteristics of study patients

Variable	DAPT group (*n* = 168)	Argatroban group (*n* = 136)	*P* Value
*Demographics*			
Age, mean (SD), y	62 ± 7.8	63 ± 6.4	0.17
Male, no. (%)	96(57.1)	73 (59.3)	0.34
*Medical history*			
Hypertension, no. (%)	91(54.1)	71(52.2)	0.73
Diabetes mellitus, no. (%)	86(51.1)	76(55.8)	0.41
Hyperlipidemia, no. (%)	93(55.3)	65(47.7)	0.88
Current smoker, no. (%)	70(41.6)	62(45.5)	0.49
History of stroke, no. (%)	22(13.0)	20(14.7)	0.62
History of coronary artery disease, no. (%)	65(38.6)	47(34.5)	0.45
*Laboratory measures*			
Glucose, mean (SD), mmol/L	7.2 (2.6)	7.4 (3.1)	0.61
Hematocrit, mean (SD)	0.41(0.16)	0.43(0.24)	0.68
Fibrinogen, mean (SD), g/l	4.3(1.5)	4.0(1.0)	0.44
WBC count, mean (SD), 10^9^/L	7.6(2.5)	7.8(2.2)	0.63
BUN, mean (SD), mmol/L	4.6(1.4)	4.7(1.6)	0.72
Cr, mean (SD), mmol/L	66(8.2)	64(6.4)	0.63
*Clinical characteristics*			
SBP, Median (IQR), mmHg	159 (125–168)	140 (121–151)	0.03
Initial diameter, mean (SD), mm	17.6 ± 2.1	17.8 ± 2.1	0.29
Presence of microbleeds, no. (%)	20(11.9)	16(13.0)	0.59
Plaques in superior side, no. (%)	25/46(54.3)	20/32(62.5)	0.49
Baseline NIHSS score, Median (IQR)	4 (3–7)	4 (3–6)	0.47
*Medicine*			
Stain, no. (%)	21(12.5)	19(13.9)	0.82
Antihypertensive, no. (%)	87(51.8)	59(43.4)	0.56

*Abbreviations*: *SD* standard deviation, *IQR* interquartile range, *NIHSS* National Institutes of Health Stroke Scale, *WBC* white blood cell, *BUN* blood urea nitrogen, *Cr* creatinine, *SBP* systolic pressure

### Primary outcome

**Fig. 3:**
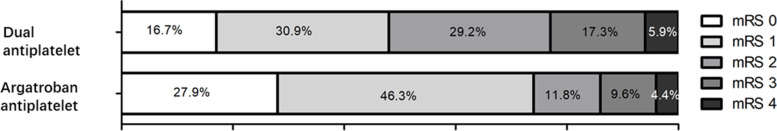
**A.** Distribution of 90-day modified Rankin scale (mRS) scores

**Fig. 4:**
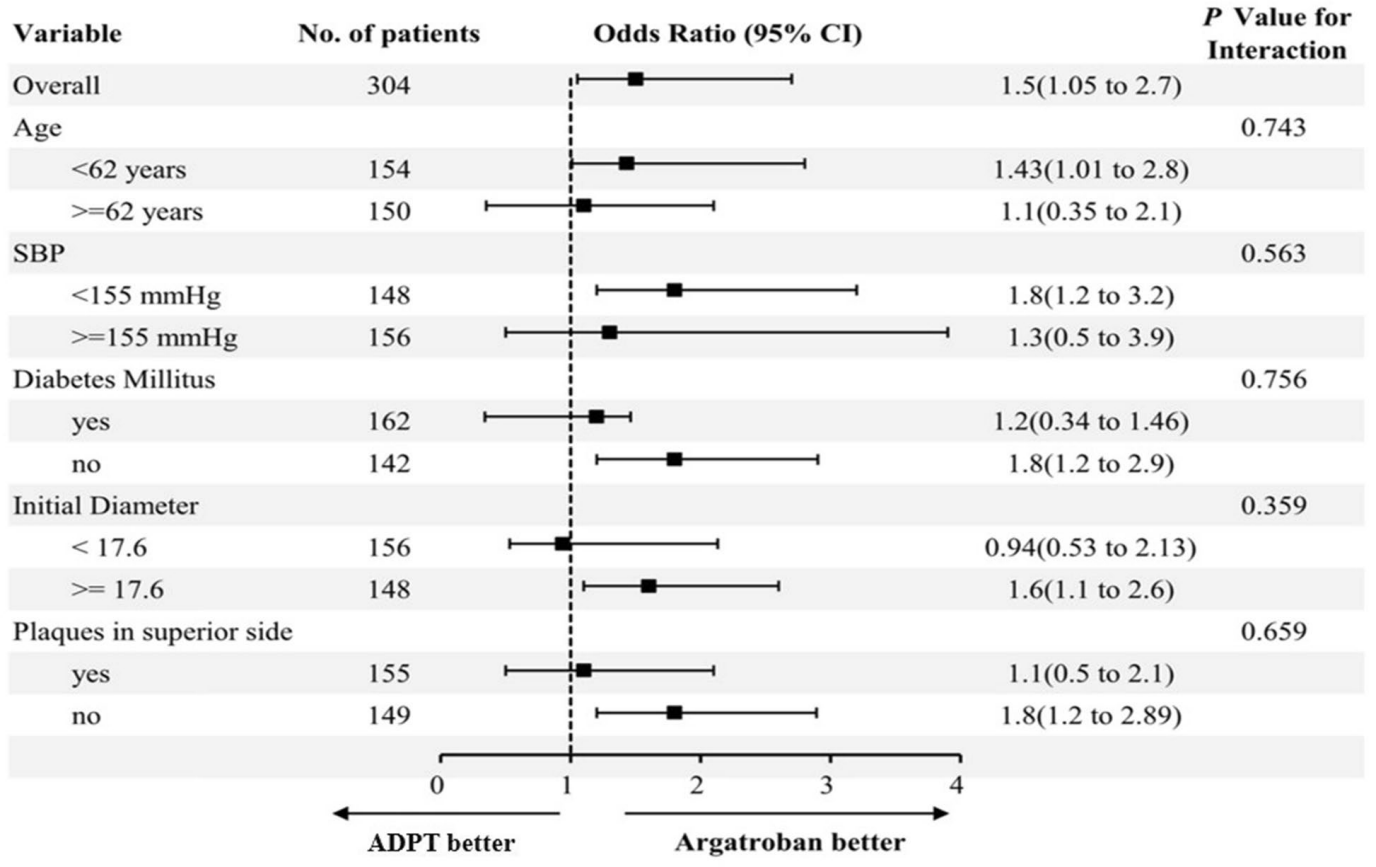
This forest plot shows that the difference in the primary clinical outcome (common odds ratio indicating the odds of improvement of 0–1 point on the modified Rankin Scale at 90 days, analyzed with the use of ordinal logistic regression) favored the argatroban group across all prespecified subgroups

In the argatroban group, 74.2% (101/136) of patients had an excellent clinical outcome (an increase from 0 to 1 in the mRS score) 3 months after stroke, significantly more than in the DAPT group (47.6%; 80/168) (*P* < 0.001) (Table 2 and Fig. 3). Binary analyses identified argatroban as an independent factor for good functional outcome (Table 3). These results suggested that argatroban with antiplatelet monotherapy was superior to the dual antiplatelet therapy. Subgroup analysis indicated that SSI patients in the argatroban group who were age<62 years, SBP<155 mmHg, no diabetes mellitus, initial diameter > = 17.6 mm and no plaques in superior side of MCA had excellent outcomes compared with those in the DAPT group (Fig. 4).

**Table 2:** Comparison of outcomes between the DAPT and argatroban groups

Variable	DAPT group (*n* = 168)	Argatroban group (*n* = 136)	*P* Value
mRS score of 0–1 at 90 days, no. (%)	80(47.6)	101(74.2)	<0.001
END, no. (%)	37(22.0)	10(7.4)	<0.001
Extracerebral bleeding, no. (%)	2(1.1)	3(2.2)	0.48
Total NIHSS score at discharge, Median (IQR)	3(2–6)	2(2–4)	0.04
Motor NIHSS at discharge, Median (IQR)	3(1–5)	2(1–3)	0.03
Sense NIHSS at discharge, Median (IQR)	1(1–2)	1(1–3)	0.57
Ataxia NIHSS at discharge, Median (IQR)	1(1–2)	1(1–2)	0.42
Hospitalization days, mean (SD), d	8.5 ± 1.9	8.0 ± 1.3	0.015
Venous thrombosis, no. (%)	18(10.7%)	8(5.8%)	0.13
APTT at 24 h, mean (SD), s	22.6 ± 3.1	41.5 ± 3.2	<0.001

*Abbreviations*: *mRS* modified Rankin Scale, *SD* standard deviation, *IQR* interquartile range, *NIHSS* National Institutes of Health Stroke Scale, *END* early neurological deterioration, *APTT* activated partial thromboplastin time

**Table 3:** Multivariable analyses of functional outcome

Variables	Odds ratio	95% Confidence interval	*P* value
Age	1.26	0.37–2.73	0.36
Diabetes mellitus	1.53	0.80–2.95	0.77
SBP	1.50	0.78–2.63	0.86
Glucose	1.45	0.71–3.18	0.62
Initial infarction diameter	1.37	0.82–2.79	0.73
Plaques in superior side	1.48	1.13–3.54	0.004
Argatroban group	1.50	1.05–2.70	0.003

*Abbreviations*: *SBP* systolic blood pressure

### Secondary outcomes

END occurred significantly more frequently in patients of the DAPT group (37; 22.0%) than in those of the argatroban group (10; 7.4%) (*P* < 0.001). These results demonstrated that early combined argatroban and antiplatelet monotherapy may attenuate progressive neurological deficits. Total NIHSS scores and motor NIHSS scores at discharge were significantly lower in the argatroban group than in the DAPT group. In addition, patients in the argatroban group had fewer hospitalization days compared with the DAPT group (*P* = 0.015). The APTT was 41.5 ± 3.2 s in the argatroban group and 22.6 ± 3.1 s in the DAPT group. However, the occurrence rate of venous thrombosis was 5.8% in the argatroban group, lower than that in the DAPT group (10.7%); although, the difference was not statistically significant (Table 2).

### Safety outcomes

ICH was not identified in either group; however, in the argatroban group, urinary hemorrhage occurred in two patients, and skin mucosal bleeding occurred in one patient after argatroban administration. The bleeding stopped after drug withdrawal. In the DAPT group, two patients developed hematochezia on days 5 and 7 after admission (Table 2).

## Discussion

In this retrospective study, we observed that early combined administration of argatroban (a DTI) and an antiplatelet agent (aspirin or clopidogrel) decreased the incidence of END and improved neurological recovery in patients with acute non-lacunar SSI associated with mild ICAS. Moreover, ICH was not identified in the argatroban group. This study provides a promising therapeutic strategy for the treatment of lon-lancular SSI associated with mild ICAS, a condition that is associated with a high prevalence of poor prognosis in many of the world’s ethnic groups.

Substantial progress has been made in the treatment of stroke with ICAS. In patients with minor ischemic stroke or high-risk TIA, those who received a combination of clopidogrel and aspirin had a lower risk of major ischemic events at 90 days [17] and that in chinese population, double antiplatelet therapy (aspirin and clopidogrel) improved neurological deficits in patients with minor strokes within 3 weeks of onset without increasing the risk for hemorrhage [7]. However, patients with proximal SSI have progressive motor deficits and unfavorable functional outcomes that are significantly associated with larger infarction volumes [6]. In addition, data indicate that patients with SSI associated with intracranial branch atheromatous disease display large infarct size, ≥3 infarct slices on serial axial diffusion-weighted imaging (DWI), progressive motor deficits, and poor functional outcomes [4, 8, 18]. Moreover, END occurs in ≥20% of SSIs and hinders functional recovery, which is relevant to arterial stenosis and branch atheromatous lesions [9]. Consistent with the above data, in our study, 22.0% of patients with nonl-lancular SSI caused by ICAS in the DAPT group experienced END within 120 h of admission with a high dependency rate 3 months after stroke onset (Table 2, Fig. 3). These observations highlight the need for the development of new therapeutic strategies for the treatment of this condition.

Although anticoagulation therapy is an option for the treatment of patients with acute ischemic stroke (AIS), related results from clinical trials have been inconclusive [19, 20]. The early direct oral anticoagulant (DOAC) treatment might be effective and acceptably safe and associated with a low frequency of clinically symptomatic intracranial haemorrhage, so its safety and efficacy need to be confirmed in randomised trials [21]. In addition, meta-analyses of completed trials have shown that anticoagulation therapy does not alter functional outcomes and that any apparent benefit in reducing early recurrence was offset by an increase in symptomatic ICH [22]. Nevertheless, guidelines have recommended the use of low-dose anticoagulants for the prophylaxis of venous thromboembolic events, even though symptomatic ICH and pulmonary embolism rates are similar with early treatment [23]. In contrast, intravenous heparin sodium administered early can be beneficial for patients with an acute hemispheric cerebral infarction, despite the increased frequency of symptomatic brain hemorrhages [24].

These results suggest that anticoagulation therapy may convey benefits for patients with ischemic stroke provided that the risk of hemorrhage is reduced. In recent decades, DTIs have been developed with efficacy comparable to that of warfarin, are associated with significantly fewer bleeding complications than warfarin, and may be effective in patients with ischemic stroke [10]. The usefulness of argatroban, dabigatran, or other thrombin inhibitors for the treatment of patients with AIS is currently not well established [14]. Further clinical trials have reported some useful information. Dabigatran treatment within 24 h of a minor stroke with no evidence of AF is feasible [25], while argatroban, which directly blocks thrombin activity without the need for the cofactor antithrombin III, consequently inhibits fibrin formation, platelet aggregation, and vascular contraction induced by thrombin [26], and has been approved in China and Japan for the management of patients with AIS. Furthermore, in patients with AIS, argatroban significantly prolongs aPTTs without increasing ICH or major bleeding events compared with heparin and warfarin for the same anticoagulant effect [11]. Our study further confirmed the above results. APTT was significantly longer in the argatroban group than in the DAPT group, and ICH was not detected (Table 2). Additionally, early-stage argatroban administration has been shown to improve neurological symptoms (motor paralysis) and daily living activities (walking, standing up, continuous sitting, and eating) [26]. Moreover, in the ARTSS-2 trial, patients with ischemic stroke associated with proximal intracranial arterial occlusion treated with r-tPA and adjunctive argatroban had excellent outcomes at 90 days that were additionally not associated with an increased risk of symptomatic ICH [12]. Even in patients treated with r-tPA and endovascular therapy (EVT), concomitant argatroban administration is feasible and safe, does not delay EVT provision, and produces high rates of recanalization [27]. Lastly, the combination of aspirin and argatroban may prove to be an effective therapeutic strategy for the prevention of coronary thrombosis [28], and argatroban in combination with antiplatelet therapy is safe in acute posterior infarction patients [29, 30]. These results suggest that argatroban administration is effective and safe for use in the treatment of ischemic stroke.

Consistent with the above studies, our research demonstrated that early combined administration of argatroban and a single antiplatelet agent decreased the incidence of END and improved neurological impairment recovery in patients with acute non-lacunar SSI associated with mild ICAS. The possible benefits include the following: In patients with SSI caused by ICAS, atherosclerotic plaques rupture, leading to secondary thrombus formation that blocks the proximal portion of a perforating artery. Argatroban can inhibit local thrombin formation in response to ischemia, prevent subsequent microthrombus formation, and improve blood flow to peri-ischemic areas, thereby rescuing at-risk neuronal cells [11]. In addition, antiplatelet agents prevent red thrombus expansion by inhibiting platelet aggregation. Thus, early combined administration of argatroban and an antiplatelet agent may prevent END occurrence and improve the recovery of neurological function. However, END within 120 h of admission was not only a reason for unfavorable outcome at 90 days, and some patients with simultaneous involvement of the posterior limb and genu of the internal capsule, corona radiata, and lentiform were likely to have unfavorable outcomes at 90 days [31].

Additionly, in our study, patients with SSI caused by ICAS were screened by brain MRI, and cerebral infarction was confirmed by DWI and apparent diffusion coefficient (ADC) sequencing. Stroke mimics were excluded. Importantly, patients with SSI caused by lipohyalinosis of a perforating artery were identified to balance the benefits and risks of anticoagulant treatment. However, there are also contradictory reports regarding the beneficial effects of argatroban administration. A retrospective observational study revealed that argatroban was safe but provided no added benefit in early outcome after acute atherothrombotic stroke [32]. There are several differences between that and the present study. First, many patients in their study underwent head CT scans, and the argatroban group may have included patients with more severe stroke compared with the DAPT group. Second, argatroban was administered beyond 1 day after onset in many patients, although earlier anticoagulation treatment may confer better outcomes [21]. Third, the study population may have included patients with ischemic stroke of a subtype other than atherothrombotic [32]. Our results also suggested that hospital stay duration was shorter in the argartrban group than in the DAPT group, which may explain the low rate of END. However, the occurrence rate of venous thrombosis did not differ between the two groups. Although the cause was not clear, it may be associated with the short duration of argatroban administration. Further research is needed to confirm this possibility.

Although effective imaging markers for SSI are lacking, with the development of MRI, MRA, and HR–MRI, several studies have proposed that lesions located in the lowest portion of the basal ganglia and the infarction volume may be suitable as imaging markers for ISS with ICAS [3, 33, 34]. In addition, HR–MRI can be used to identify plaque extension over small penetrating artery ostia, which can result in SSI [35, 36]. Similar to the above results, our study showed that in patients with acute non-lacular SSI associated with mild ICAS, the initial infarct diameter reached 17.8 ± 2.1 and 17.6 ± 2.1 mm, the number of infarct imaging slices ranged from 3 to 5, and the lesion location extended to the basal surface of the MCA on DWI (Fig. 2). Moreover, HR–MRI results demonstrated the presence of atherosclerotic plaques on the upper wall of the MCA (Fig. 2). Therefore, the use of MRI and HR–MRI techniques in the assessment of SSI may aid in better delimiting the boundaries of branch atheromatous disease as a nosological entity [37].

Of course, our study also has limitations. First, this is a small-scale retrospective observational research that might produce assessment bias. Second, only two-thirds of Grade hospital attended the study. Third, a placebo control group is lacking. Therefore, a multicenter, double-blind, placebo control study with larger sample sizes should be conducted.

## Conclusions

In conclusion, in this study, we observed that early combined administration of argatroban and a single antiplatelet agent (aspirin or clopidogrel) could be beneficial for patients with acute non-lacunar SSI associated with mild ICAS screened by MRI. Furthermore, this combination could attenuate progressive neurological deficits and did not cause cerebral hemorrhage. This was a retrospective study and further prospective, multicenter, double-blind, placebo-controlled studies with large sample sizes should be performed to confirm these findings.

## Supplementary Information

**Additional file 1 **: **Supplementary Table S1.** Baseline characteristics of study patients.

## Data Availability

The datasets used or analysed during the current study are available from the corresponding author on reasonable request.

## Abbreviations

SSI: Single subcortical infarction
ICAS: Intracranial atherosclerosis
END: Early neurological deterioration
DAPT: Dual antiplatelet therapy
ICH: Intracranial hemorrhage
DTIs: Direct thrombin inhibitors
r-tPA: Recombinant tissue plasminogen activator
NIHSS: National Institutes of Health Stroke Scale
mRS: Modified Rankin scale
MCA: Middle cerebral artery
SSS-TOAST: The Stop Stroke Study Trial of Org 10,172 in Acute Stroke Treatment
MRA: Magnetic resonance angiography
AF: Atrial fibrillation
APTT: Activated partial thromboplastin time
MRI: Magnetic resonance imaging
3D TOF–MRA: Three-dimensional time-of-flight MRA
IQR: Interquartile range
WBC: White blood cell
SBP: Systolic blood pressure
DWI: Diffusion-weighted imaging
AIS: Acute ischemic stroke
ADC: Apparent diffusion coefficient
DOAC: Direct oral anticoagulant
