# Supplementary material for: Early argatroban and antiplatelet combination therapy in acute non-lacunar single subcortical infarct associated with mild intracranial atherosclerosis
Source: BMC Neurol. 2021 Nov 10;21:440. doi: 10.1186/s12883-021-02435-x (PMC8579679; doi:10.1186/s12883-021-02435-x)
Supplement: Supplementary file 1 — Additional file 1 : Supplementary Table S1. Baseline characteristics of study patients. [file 12883_2021_2435_MOESM1_ESM.docx]

**Supplementary Table S1. Baseline characteristics of study patients.**

| variable | mRS 0–1  (n=181) | mRS 2–6  (n=123) | *P* Value |  |
| --- | --- | --- | --- | --- |
| *Demographics* |  |  |  |  |
| Age, mean (SD), y | 60.7 (12.6) | 67.5 (11.5) | 0.04 |  |
| Male, no. (%) | 108 (59.6) | 73 (59.3) | 0.72 |  |
| *Medical history* |  |  |  |  |
| Hypertension, no. (%) | 92 (50.8) | 70(56.9) | 0.21 | |
| Diabetes mellitus, no. (%) | 80 (44.2) | 82 (66.7) | 0.03 | |
| Hyperlipidemia, no. (%) | 94 (51.9) | 64 (52.0) | 0.88 | |
| History of stroke, no. (%) | 24 (13.3) | 18 (14.6) | 0.62 | |
| History of coronary artery disease, no. (%) | 66 (36.4) | 46 (37.4) | 0.49 | |
| *Laboratory measures* |  |  |  | |
| Glucose, mean (SD), mmol/L | 6.9 (2.5) | 8.3 (3.9) | 0.01 | |
| Hematocrit, mean (SD) | 0.42(0.15) | 0.44(0.22) | 0.58 | |
| Fibrinogen, mean (SD), g/l | 4.2(1.4) | 3.9(1.2) | 0.67 | |
| WBC count, mean (SD), 10^9^/l | 7.8(2.4) | 7.5(2.0) | 0.57 | |
| BUN, mean (SD), mmol/L | 4.5(1.3) | 4.8(1.5) | 0.64 | |
| Cr, mean (SD), mmol/L | 68(7.9) | 64(7.4) | 0.55 | |
| *Clinical characteristics* |  |  |  | |
| SBP, Median (IQR), mmHg | 140 (124–158) | 156 (133–165) | 0.04 | |
| Initial diameter, mean (SD), mm | 15.2(1.2) | 18.6(1.1) | 0.02 | |
| Presence of microbleeds, no. (%) | 23 (12.7) | 16(13.0) | 0.59 | |
| Plaques in superior side, no. (%) | 12(6.6) | 33(26.8) | 0.02 | |
| Baseline NIHSS score, Median (IQR) | 4 (2–5) | 4 (3–6) | 0.85 | |
| *Medicine* |  |  |  | |
| Stain, no. (%) | 20(11.0) | 16(13.0) | 0.87 | |
| Antihypertensive, no. (%) | 89(49.2) | 59(47.9) | 0.85 | |
| Weihai Municipal Hospital, no. (%) | 99(54.7) | 65(52.8) | 0.80 | |
| Argatroban group, no. (%) | 101(55.8) | 35(28.5) | 0.01 | |

Abbreviations: SD = standard deviation; IQR = interquartile range; NIHSS = National Institutes of Health Stroke Scale; WBC = white blood cell; BUN = blood urea nitrogen; Cr = creatinine; SBP = systolic pressure.
